# Critical role of NLRP3 in causing paravertebral muscle injury in adolescent idiopathic scoliosis

**DOI:** 10.1002/ctm2.1528

**Published:** 2024-02-08

**Authors:** Zhuo‐Tao Liang, Jiong Li, Hao Tang, Jia‐Ke Li, Meng‐Jun Li, Chao‐Feng Guo, Hong‐Qi Zhang

**Affiliations:** ^1^ National Clinical Research Center for Geriatric Disorders Xiangya Hospital Central South University Changsha Hunan P.R. China; ^2^ Department of Spine Surgery and Orthopaedics Xiangya Hospital, Central South University Changsha Hunan P.R. China; ^3^ Department of General Surgery The Third Xiangya Hospital, Central South University Changsha Hunan P.R. China

Dear Editor,

During the development and treatment of adolescent idiopathic scoliosis (AIS), obvious soft tissue injury around the vertebrae and back pain are always present.^1^, ^2^ Abnormal paraspinal muscle development can affect the progression and rehabilitation of scoliosis.^3^ However, there is insufficient attention to the pathogenesis and personalised treatment for addressing the abnormal changes in the paraspinal muscles in patients with AIS. Our study found that the activation of the NLRP3/GSDMD pathway, a key factor in paraspinal muscle injury of patients with AIS, and a nano‐drug delivery system we designed can effectively inhibit this damage.

In this study, we enrolled a total of 30 patients with AIS and 15 age‐ and gender‐matched patients with non‐AIS (Table S1). Clinical imaging analysis revealed high levels of fat infiltration in AIS paraspinal muscle, but no significant differences were observed between concave and convex sides (Figure S1A,B). Next, we investigated anomalous changes in the paravertebral muscle tissues of patients with AIS, including increased muscle cell pyroptosis and apoptosis, increased inflammatory infiltration and decreased proliferation ability of primary paravertebral muscle cells (Figures 1A–C and S1). Subsequently, RNA sequencing was performed on paravertebral muscle tissues to identify essential genes associated with AIS paraspinal muscle injury. Through various bioinformatic analyses and qPCR validation, we identified *NLRP3*, an important regulator in pyroptosis pathway,^4^ as a key candidate gene (Figures 1D–G and S2A,B). The sequences of all qPCR primers are listed in Table S2. Subsequently, various validation confirmed that the pyroptosis and apoptosis pathway proteins in paravertebral muscle of AIS were significantly higher than those in non‐AIS (Figures 1H–K and S2C–E). Then, we overexpressed *NLRP3* in C2C12 cells using lentiviral transfection and found that not only the NLRP3/GSDMD/IL‐1β signalling pathway was activated (Figures 2A–F and S3A–C), but also the apoptosis pathway was upregulated (Figures 2E–H and S3D–F). Meanwhile, knocking down *NLRP3* expression in primary paravertebral muscle cells of AIS using siRNA transfection significantly inhibited cell pyroptosis and apoptosis (Figure S4). The sequences of all interfering RNAs are presented in Tables S3 and S4. Some studies have reported that the N‐terminal peptides of GSDM protein families, such as GSDMD/GSDME, can activate apoptotic pathway through entering the mitochondrial.^5^

**FIGURE 1:**
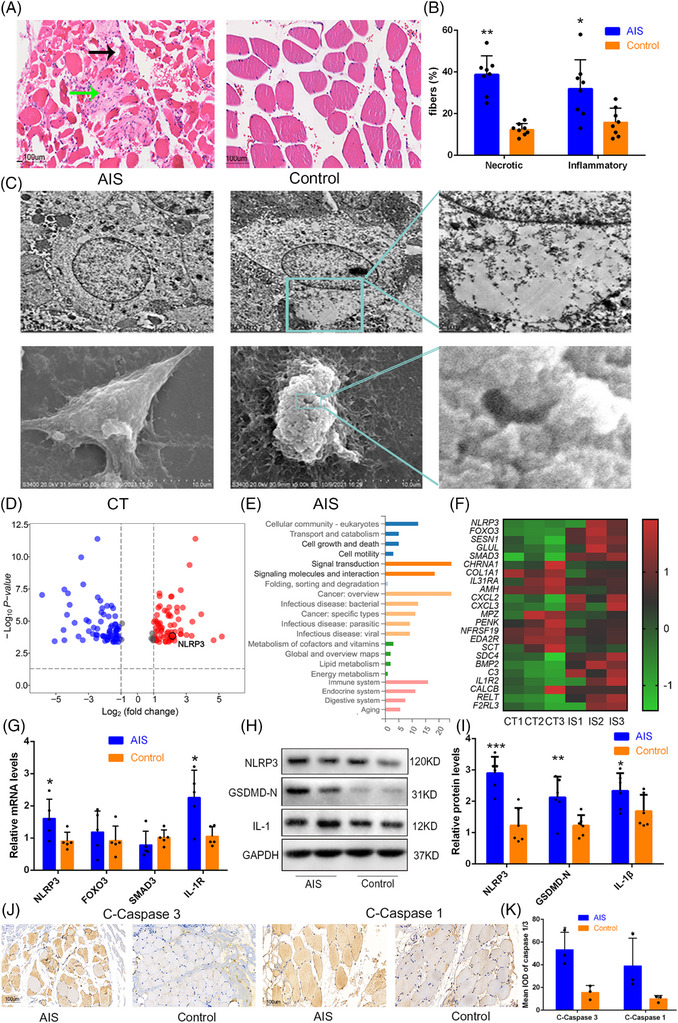
Abnormal development of paravertebral muscle in patients with adolescent idiopathic scoliosis (AIS). (A and B) Haematoxylin and eosin (HE) staining showed the proportion of inflammatory infiltration and muscle fibre necrosis in paraspinal muscle of AIS and non‐AIS. Scale bar, 100 μm, *n* = 8. (C) Representative images of pyroptosis in primary paraspinal muscle cells of AIS and non‐AIS detected by transmission electron microscopy (TEM) and scanning electron microscopy (SEM). Scale bar, 2 μm. (D) Volcano map showed the results of RNA sequencing of paraspinal muscles in patients with AIS and non‐AIS, and a total of 156 differential expressed genes (DEGs) were found (77 increased and 79 decreased). (E) Kyoto Encyclopedia of Genes and Genomes (KEGG) enrichment analyses based on these DEGs. (F) The heatmap showed a total of 23 DEGs in the cell and growth–death and signalling and molecules–interaction signalling pathway. (G) mRNA levels of four genes in paraspinal muscle tissue were verified by qPCR, *n* = 5. (H and I) Levels of pyroptosis pathway related proteins in paraspinal muscle cells from patients with AIS and non‐AIS, *n* = 6. (J and K) Immunohistochemical staining for C‐Caspase 1 and C‐Caspase 3. Scale bar, 100 μm, *n* = 3. Data are shown as the mean ± standard deviation (SD), ^*^ *p* < .05 versus the control group, ^**^ *p* < .01 versus the control group and ^***^ *p* < .001 versus the control group.

**FIGURE 2:**
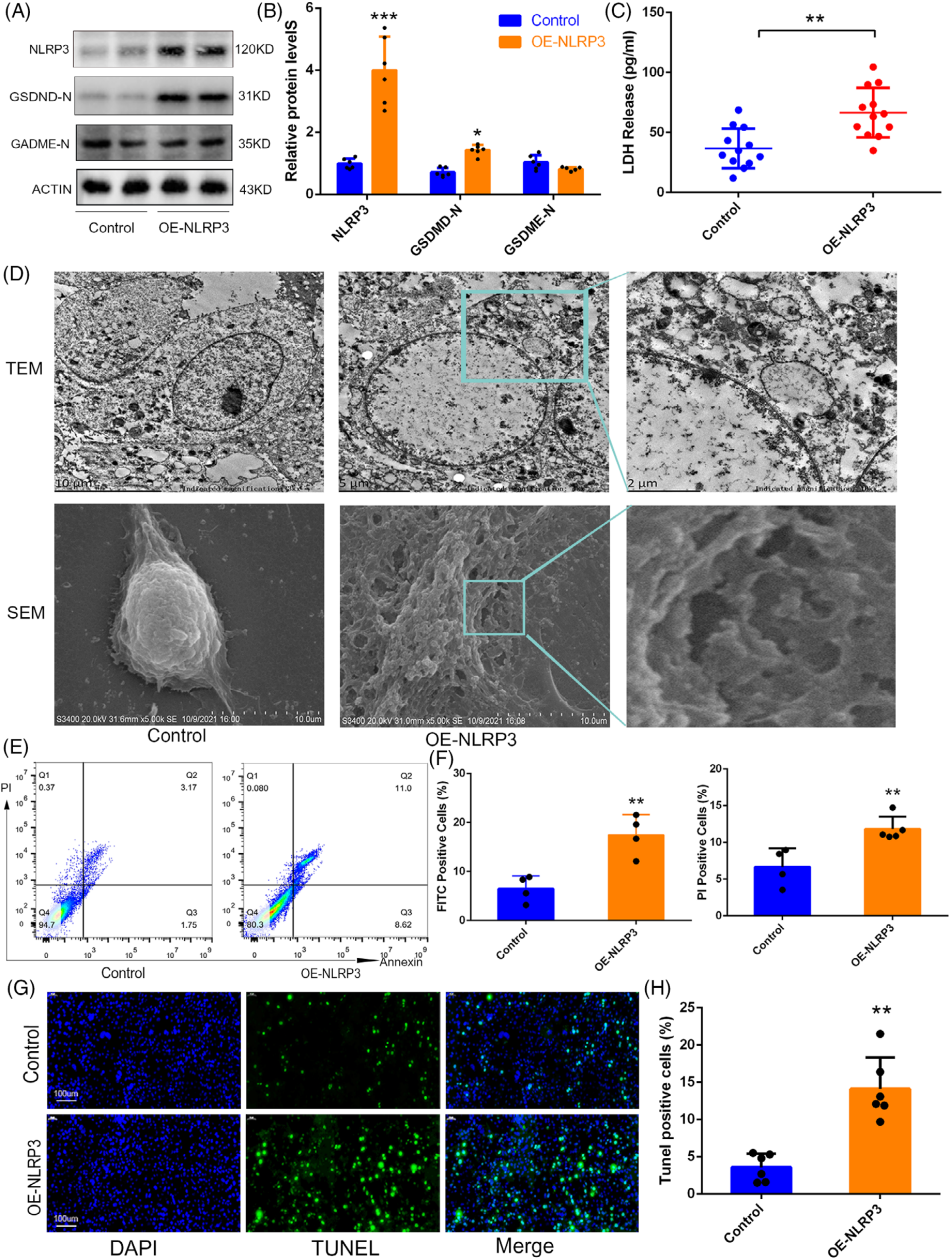
Overexpression of NLRP3 caused increased pyroptosis and apoptosis in c2c12 muscle cells. (A and B) Protein levels of NLRP3, GSDME‐N and GSDMD‐N in control and OE‐NLRP3 cells, *n* = 6. (C) Level of Lactate Dehydrogenase (LDH) in the supernatant of culture medium from normal and OE‐NLRP3‐treated cells, *n* = 12. (D) Representative images of pyroptosis in control and OE‐NLRP3 c2c12 cells detected by transmission electron microscopy (TEM) and scanning electron microscopy (SEM). Scale bar, 2 μm. (E and F) The proportion of Fluorescein Isothiocyanate (FITC) and Propidium Iodide (PI) positive cells in normal and OE‐NLRP3‐treated group, *n* = 4. (G and H) Representative images of TUNEL immunofluorescence staining in normal and OE‐NLRP3 groups. Scale bar, 100 μm, *n* = 6. Data are shown as the mean ± standard deviation (SD), ^*^ *p* < .05, **p＜.01 and ^***^ *p* < .001 versus the control group.

Therefore, we further explored the mechanism by which *NLRP3* simultaneously activates pyroptosis and apoptosis in AIS paraspinal muscle cells. Using Crispr‐Cas9 technology to knockout *GSDMD* expression in C2C12 cells, we found that both the activation of pyroptotic and apoptotic pathway caused by OE‐NLRP3 were inhibited (Figures S5 and S6). Moreover, JC1 staining suggested that GSDMD‐N activated the cell apoptosis by affecting the mitochondrial membrane potential and increasing the expression of CYTC (Figure 3D–G). In addition, novel material‐based drugs have been demonstrated to have promising applications in many diseases, such as aneurysm.^6^, ^7^ Here, we designed a nanoparticle (NP) drug containing disulphiram (DSF), a recognised inhibitor of GSDMD.^8^ The NP we constructed contains thioketal bonds, which would break and release DSF under oxidative stress stimulation.^9^ Through multiple analyses, we examined a variety of physicochemical properties of NP (Figure S7).

**FIGURE 3:**
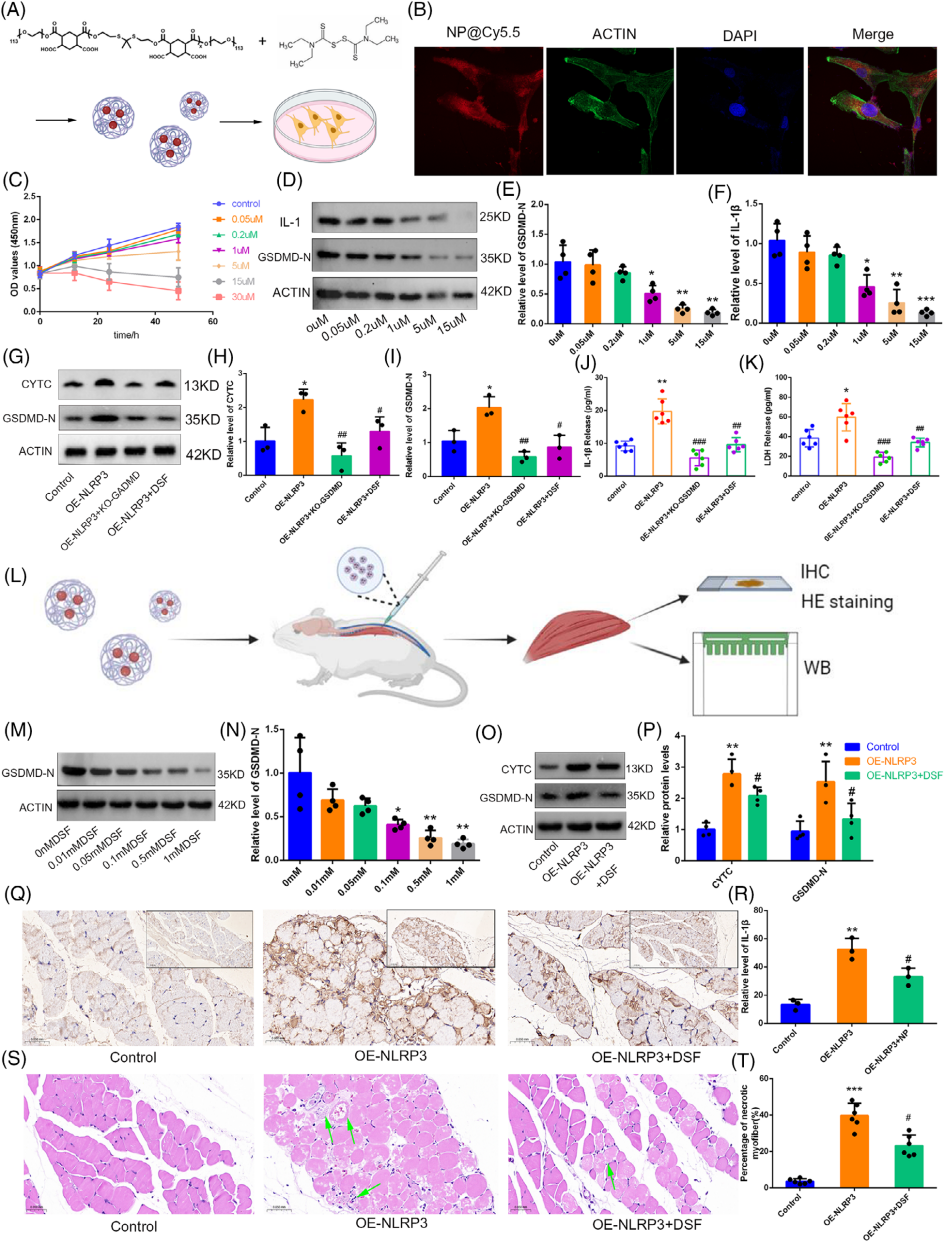
Nanoparticle (NP) can effectively reduce the pyroptosis and apoptosis caused by NLRP3 elevation via inhibiting the formation of GSDMD‐N. (A) The composition of NP and the schematic diagram of cell intervention. (B) Representative immunofluorescence images showed that NPcy5.5 had been effectively endocytosed by cells Scale bar, 20 μm. (C) Proliferation of adolescent idiopathic scoliosis (AIS) primary paraspinal muscle cells were detected by Cell‐Counting‐Kit‐8 (CCK8). It showed that cell proliferation gradually stopped when the concentration of NPs exceeded 5 μM. (D–F) Western blotting (WB) showed that when the concentration of NPs exceeded 1 μM, the expression levels of GSDMD‐N and interleukin‐1β (IL‐1β) protein were significantly reduced, *n* = 4. (G–K) WB and ELISA‐Enzyme linked immunosorbent assay (ELISA) results showed that although the inhibitory efficiency of NP treatment was lower than that of GSDMD knockout, it also significantly reduced the pyroptosis and apoptosis caused by NLRP3 overexpression. (L) Schematic diagram of the efficacy evaluation of NP in vivo. (M and N) We set up a concentration gradient to explore the appropriate intervention conditions; analysis showed that when the concentration of NP exceeded .1 mM, the levels of GSDMD‐N protein in muscle tissue was significantly reduced, *n* = 4. (O and P) Subsequently, we treated mice with NP and set up control, OE‐NLRP3 and OE‐NLRP3 + NP groups. WB results showed that NP treatment significantly inhibited the protein levels of GSDMD‐N and CYTC, *n* = 4. (Q and R) Representative images showing immunohistochemical staining for IL‐1β. Scale bar, 100 μm, *n* = 3. (S and T) Haematoxylin and eosin (HE) staining showed that overexpression of NLRP3 increased muscle tissue necrosis, but NP treatment rescued muscle fibre necrosis, at least in part, *n* = 6. Data are shown as the mean ± standard deviation (SD), ^*^ *p* < .05 **p < .01 and ^***^ *p* < .001 versus the control group, ^#^ *p* < .05, ^##^ *p* < .01 and ^###^ *p* < .001 versus the OE‐NLRP3 group.

In vitro experiments showed that it could be effectively taken up by muscle cells (Figure 3A,B), and could effectively inhibit the pyroptosis and apoptosis of AIS primary paravertebral muscle cells at appropriate doses (Figure 3C–K). In vivo study, OE‐NLRP3 and OE‐NLRP3 + NP groups were established via paraspinal muscle injection adenovirus or NP. The results suggested that at the appropriate concentration, NP could effectively inhibited the activation of the pyroptosis pathway and the necrosis of paraspinal muscle fibres caused by OE‐NLRP3 (Figure 3L–T). Figure 4 is the schematic diagram of the critical role of NLRP3 in causing paravertebral muscle Injury in patients with AIS.

**FIGURE 4:**
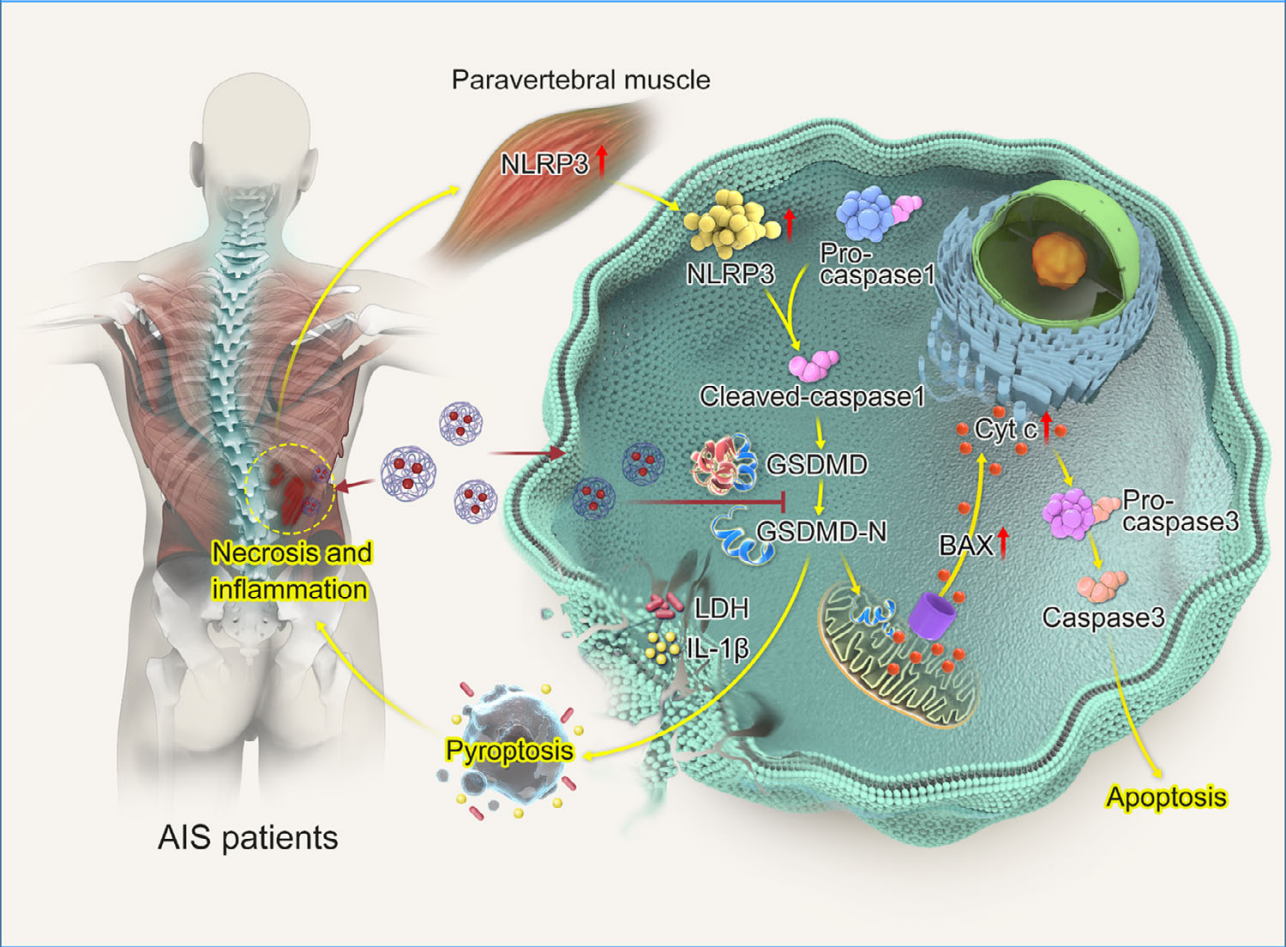
The schematic diagram of the critical role of NLRP3 in causing paravertebral muscle injury in patients with adolescent idiopathic scoliosis (AIS).

In conclusion, we first demonstrated that the high level of NLRP3 in AIS mediates severe inflammatory events, further inducing pyroptosis and apoptosis through the activation of GSDMD, which is an important pathogenic factor in paraspinal muscle injury in AIS. Moreover, the use of NP can effectively alleviate the paraspinal muscle injury caused by OE‐NLRP3, offering potential targets for effective and personalised clinical treatment of AIS and providing new insights for the development of novel drugs.

## AUTHOR CONTRIBUTIONS

*Conceived and completed the experiment*: Zhuo‐Tao Liang, Hao Tang and Meng‐Jun Li. *Data analysis and interpretation*: Jia‐Ke Li. *Assembly and collection of data*: Jiong Li and Zhuo‐Tao Liang. *Administrative and financial support*: Hong‐Qi Zhang and Chao‐Feng Guo. *Manuscript writing*: Zhuo‐Tao Liang. *Guidance for writing and submission*: Hong‐Qi Zhang and Chao‐Feng Guo.

## CONFLICT OF INTEREST STATEMENT

The authors declare they have no conflicts of interest.

## ETHICS STATEMENT

The human sample study and animal study were reviewed and approved by the Ethics Committee approval of Xiangya Hospital. Informed written consent was given by all the participants and their legal guardians before study.

## Supporting information

Supplementary Methods

Supplementary Figures

Supplementary Tables
