# Supplementary material for: Critical role of NLRP3 in causing paravertebral muscle injury in adolescent idiopathic scoliosis
Source: Clin Transl Med. 2024 Feb 8;14(2):e1528. doi: 10.1002/ctm2.1528 (PMC10851084; doi:10.1002/ctm2.1528)
Supplement: Supplementary file 1 — Supplementary Methods [file CTM2-14-e1528-s003.docx]

**Materials and methods**

**Subjects**

We recruited 30 AIS patients between 10 and 18 years-of-age and 15 sex- and age-matched patients without AIS (Table s1). The inclusion criterion used for AIS patients was a minimal Cobb angle greater than 60°; and ruled out scoliosis caused by other diseases including congenital spinal deformity, Marfan syndrome, neuromuscular diseases or spinal deformities caused by other syndromes through physical examination and imaging. The inclusion criteria for the control group were age-matched non-AIS patients, including healthy individuals and patients with lumbar herniation or spine fracture at our medical examination center. The patients in the control group were subjected to a comprehensive X-ray examination to confirm that no spinal deformity existed.

**In vivo assay**

All animal experiments were reviewed and approved by the Animal Care and Use Committee of the Laboratory Animal Research Center at Xiangya Medical School of Central South University. A total of 18 three-week-old female c57 mice were purchased from the Central South University Animal Department (Changsha, China). Mice were divided into three groups: control group (n=6), OE-NLRP3 group (n=6), and OE-NLRP3+NP group (n=6). Adenovirus were injected into the paraspinal muscle tissue to over-express *NLRP3* at week 3 and 4, and saline injected mice served as controls. Then, mice in OE-NLRP3+NP group were injected with 0.1mM NP into the paraspinal muscles three times at week 6, 8, and 10, and the other two groups were injected with saline. Peripheral blood and paraspinal muscle tissues were collected for further analysis.

**RNA extraction and RNA-seq**

Total RNA was extracted from fresh-frozen paravertebral muscle tissue samples using TRIzol reagent (Invitrogen, California, USA). Total RNA quality and quantity were determined using a Nanodrop 8000 UV-Vis spectrometer (Thermo Scientific Inc, USA). RNA-seq was performed using total RNA samples with a quantity ≥ 10 μg and an RNA integrity number > 6.0. Library construction and sequencing were carried out on BGISEQ-500 by Beijing Genomic Institution (www.genomics.org.cn, BGI, Shenzhen, China). Original sequence data has been submitted to the database of NCBI Sequence Read Archive (http://trace.ncbi.nlm.nih.gov/Traces/sra). In gene expression analysis, the matched reads were calculated and normalized to RPKM using RESM software. The significance of the differential expression of genes was defined by the bioinformatics service of BGI (ccording to the combination of the absolute value of log2- Ratio ≥ 1and FDR ≤ 0.01). Gene Ontology (GO) and KEGG metabolic pathways annotation and enrichment analyses were conducted on NCBI databases: (http://www.geneontology.org/) and (http://www.genome.jp/kegg/) respectively.

**Quantitative real-time PCR (qRT-PCR)**

The experimental method used for WB was based on a previously described method[1]. The following primers were listed in Table s2.

**Western blotting (WB)**

The experimental method used for WB was based on a previously described method[1]. The following primary antibodies were used: β-actin (1:5000; Cell Signaling Technology, Boston, USA), NLRP3 (1:500; Abcam, USA), GSDMD-N (1:500; London, UK), IL-1β (1:800; CST, USA), C-Caspase 1,3 (1:1000; CST, USA), BAX (1:1000; CST, USA), BCL-2 (1:1000; CST, USA) and CYTC (1:1000; CST, USA). The membranes were subsequently incubated with secondary antibodies (1:10000, Proteintech, Shanghai, China) at room temperature for 1 h.

**Immunofluorescence (IF)**

Cells were placed on slides and fixed with 4% paraformaldehyde for 15 min, incubated with 0.3% triton solution for 10 min, sealed with 5% BSA for 30 min and then incubated with Ki67 (1:500; CST, USA) and CYTC (1:300; CST, UK) primary antibody at 4℃ overnight. The following morning, the cells were incubated with a fluorescence secondary antibody (1:300, Abcam, UK) at room temperature for 1h.

**SiRNA transfection**

First, the cells were seeded into 24-well plates at a density of 1 ×10^4^ cells per well. The cells were then transfected with lentivirus using riboFECT^TM^ CP reagent (RiboBio, Guangzhou, China) with a final concentration of 50nM in accordance with the manufacturer’s recommended protocols. The siRNA sequences used to knock-down NLRP3 are shown in Table s3. Forty-eight hours after transfection, the expression levels of NLRP3 protein were verified by western blotting.

**CRISPR-Cas9 gene knockout**

C2C12 cells were inoculated into 24-well plates and 25µl of cell culture medium was added into a microcentrifuge tube, then the Lipofectamine™ Transfection Reagent (Thermo Scientific, USA) was added to the tube. incubated at room temperature for 10min. After that, we took another centrifuge tube and added OPti-MEM^TM^ serum medium (Gibco, New York, USA), Cas9 protein (NEB, California, USA), tracrRNA (10μM) and crRNA (10μM). The mixture of crRNA, tracrRNA and Cas9 protein was then added to the diluted transfection reagent and incubated at room temperature for 5min. Following incubation, the transfection complex was added into the cell culture medium and cultured for 48 - 72 hours. The crRNA sequences designed in this experiment are listed in Table s4. Genomic DNA was then extracted and gene editing efficiency was detected using riboEDIT^TM^ T7EI Enzyme (NEB, USA). The edited cells were digested with 0.25% trypsin and the cell concentration was adjusted to 5 cells/ml. The cells were then spread on to 96-well plates (100μL per well). The 96-well plates were then cultured in an incubator for 7-14 days until the formation of monoclonal cells could be seen under a microscope at a magnification of 4X. Wells containing only one monoclonal cell group were selected and the remaining multiple clones or cell-free wells were removed. Following removal, the selected cells were digested and transferred to a 24-well plate for further culture.

**Scanning electron microscopy** (**SEM) and Transmission electron microscope (TEM)**

Cell culture medium was discarded and fixed at 4℃ with electron microscope fixative solution for 2 - 4h. Next, 1% osmium was dissolved in PBS and used to fix the cells for 2h at room temperature. Next, the cells were rinsed three times in PBS (15min per wash). The cells were dehydrated successively with 50%-70%-80%-90%-100%-100% alcohol followed by 100% acetone-100% acetone (15min each). For embedding, polymerization was performed in an oven at 60℃ for 48h. Ultra-thin slices (60-80nm) were then prepared and stained with uranium-lead double staining. TEM (Hitachi, Tokyo, Japan) and SEM (Hitachi, Tokyo, Japan) were used for observation and image analysis.

**Histological analysis and pathological valuation**

Paravertebral muscle tissues were routinely embedded in paraffin and cut into sections (5µm thick). Sections were then stained with hematoxylin-eosin (H&E). Images of 3-4 different fields per sample were then taken under a microscope equipped with a digital CCD. Semi-quantitative analyses of muscle histology were performed in a blinded fashion.

**JC-1 detection**

The JC-1 detection reagent is purchased from Beyotime Biotechnology, China, and the working liquid is configured according to the manual. Then a 0.5ml JC-1 staining solution was added to the 24-well plate cells and incubated at 37 °C in the cell incubator for 20 minutes. Followed by washing with JC-1 staining buffer for 2 times, then added 1ml JC-1 staining buffer (1x) and observed cells with a fluorescence microscope.

**Synthesis of Polymers**

The method used to synthesize polymers was described previously [2]. In brief, 2,2'-(propane-2, 2-diylbis (sulfadiyl)) -diethylene glycol and 1,2,4,5-cyclohexanecarboxylic dianhydride were dissolved in anhydrous N-dimethylformamide. After magnetic stirring at 50℃ for 24 h, mPEG5000 was added to the reaction system; the solution was then stirred for 24 h. The mixture was then placed into a dialysis bag for 48 h of dialysis. Then, the polymer was extracted by decompression freeze-drying.

2,2'-(Propane-2, 2-diyl bis (sulfadiyl)) -diethylene glycol, 1,2,4,5-cyclohexane tetracarboxylic acid dianhydride, N, and n-dimethylformamide were purchased from Aladdin (Shanghai, China). mPEG5000 and DSPE-mPEG2000 were purchased from Aladdin Reagent Co., Ltd. (Shanghai, China). DMSO was purchased from Amresco (Beijing, China). The TEM (American FEI Tecnai F20) was used to detect the morphology of NP. We also used a nanoparticle and zeta potential analyzer (DLS) (British Pennsylvania-based firm Zetasizer Nano ZS90) to measure physical characteristics of NP. Besides, nuclear magnetic resonance (NMR) spectroscopy, NMR (liquid) (German Bruker Avance NEO), and Liquid Chromatograph Mass Spectrometer (LC-MS, America's Ultimate 3000 UHPLC - Q Exactive) experiments were performed.

**Construction of nanoparticles**

2 mg of DSF was fully dissolved with 20 mg of macromolecules in 2 mL DMSO and subsequently added by titration into 20 mL of ultrapure water; this was followed by cortex mixing. After 24 hours of dialysis, the liquid in the dialysis bag was collected and filtered through a 0.45-micron filter for storage.

**Statistical analysis**

All statistical analyses were conducted using GraphPad Prism 7 (GraphPad Software, Inc., La Jolla, CA, USA) and all experiments were repeated independently three times. All data are expressed as mean ± standard deviation (SD). The K-s test was used to detect whether the data conformed to a normal distribution. A P value (sig 2-tailed) > 0.05 showed that the data conformed to a normal distribution. The student’s t-test (unpaired or paired) was used to determine the significance of differences between the two groups. Analysis of variance (ANOVA) was used to determine the significance of differences between multiple groups. Nonparametric test was used if the data did not conform to the normal distribution. P < 0.05 was considered statistically significant.

1. Rong R, Xia X, Peng H, Li H, You M, Liang Z, et al. Cdk5-mediated Drp1 phosphorylation drives mitochondrial defects and neuronal apoptosis in radiation-induced optic neuropathy. Cell death & disease. 2020;11(9):720.

2. Ding F, Li F, Tang D, Wang B, Liu J, Mao X, et al. Restoration of the Immunogenicity of Tumor Cells for Enhanced Cancer Therapy via Nanoparticle-Mediated Copper Chaperone Inhibition. Angewandte Chemie (International ed in English). 2022;61(31):e202203546.
