# Supplementary material for: Critical role of NLRP3 in causing paravertebral muscle injury in adolescent idiopathic scoliosis
Source: Clin Transl Med. 2024 Feb 8;14(2):e1528. doi: 10.1002/ctm2.1528 (PMC10851084; doi:10.1002/ctm2.1528)
Supplement: Supplementary file 2 — Supplementary Figures [file CTM2-14-e1528-s002.docx]

Figure S1


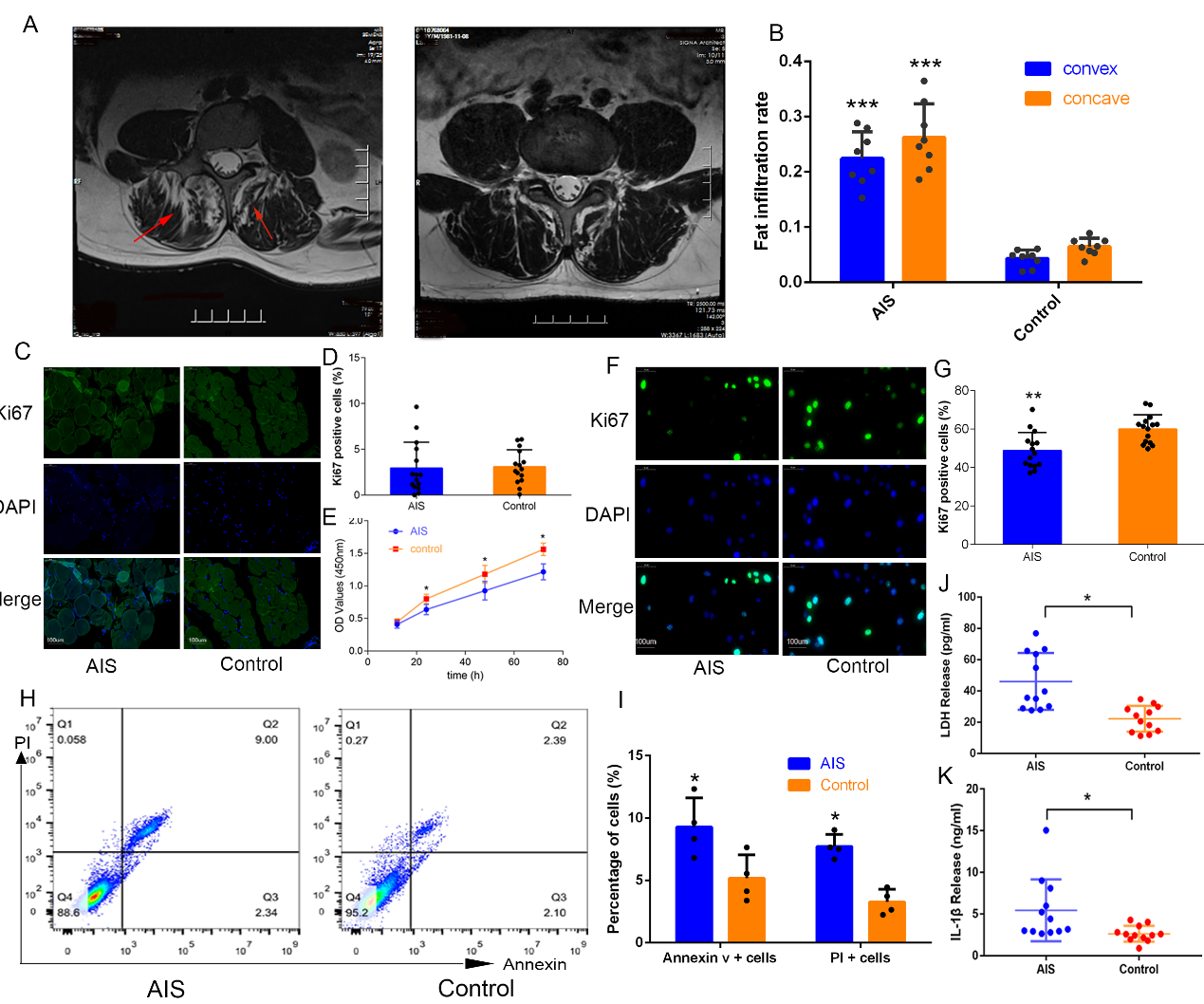


Figure s1: Anomalous changes in the paravertebral muscle of patients with AIS. (A, B) Preoperative MRI examination of patients with AIS and non-AIS, n= 8. (C, D) Representative images of Ki67 immunofluorescence staining of paraspinal muscle from patients with AIS and non-AIS. Scale bar, 100μm, n=15. (E) Detection of primary paraspinal muscle cells proliferation in patients with AIS and non-AIS by CCK8, n=10. (F, G) Representative images of Ki67 immunofluorescence staining of primary paraspinal muscle cells, Scale bar, 20μm, n=15. (H, I)The proportion of apoptosis and necrosis in primary paraspinal muscle cells of patients with AIS and non-AIS, n=4. (J, K) Levels of LDH and IL-1β in the culture medium from primary paraspinal muscle cells of patients with AIS and non-AIS, n=12. Data are shown as the mean ± SD, * means p < 0.05 vs. the control group. ** means p < 0.01 vs. the control group.

Figure S2


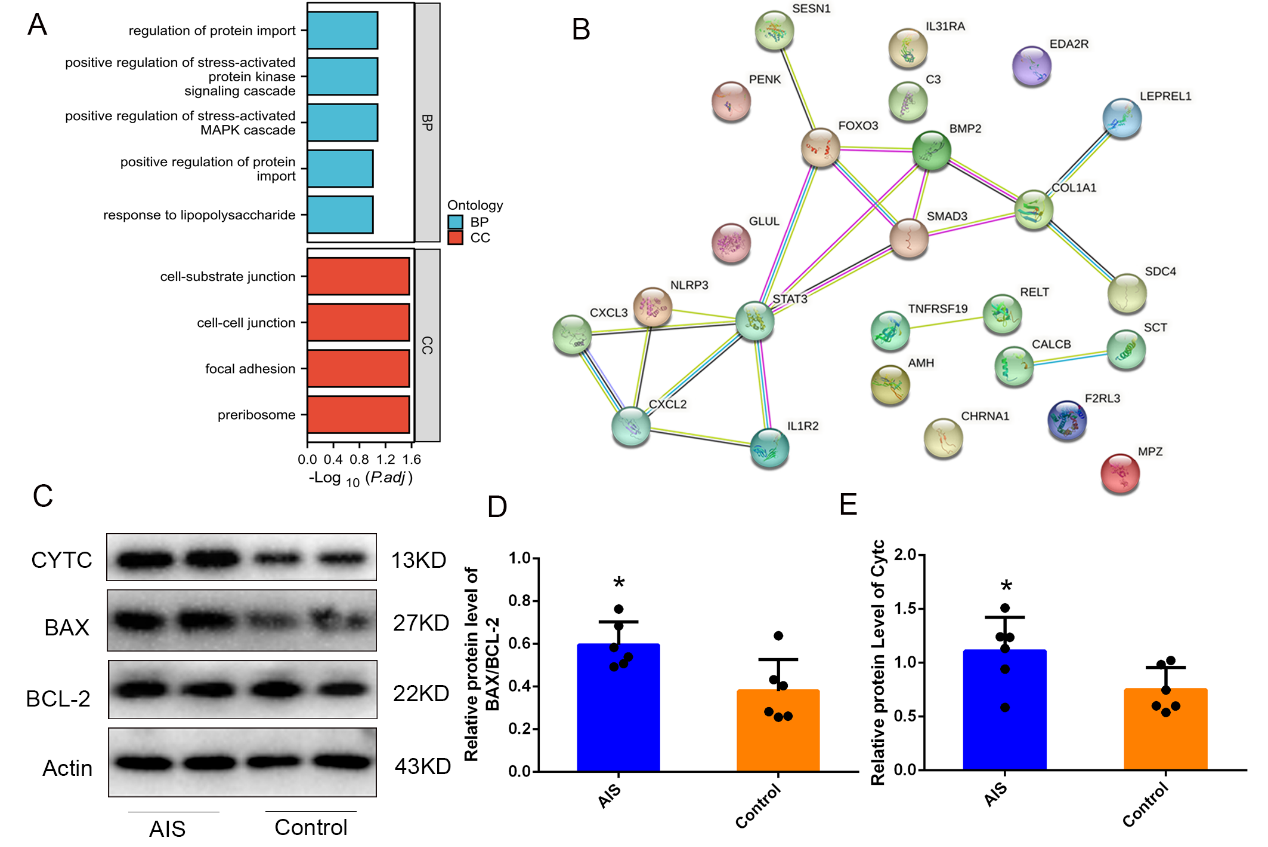


Figure s2: (A) GO enrichment analyses based on 156 DEGs. (B) PPI network representing the proteins encoded by the 23 DEGs using the STRING database. (C-E) Relative level of proteins from apoptotic pathway in patients with AIS and non-AIS, n=6. Data are shown as the mean ± SD, * means p < 0.05 vs. the control group, (n=6).

Figure S3


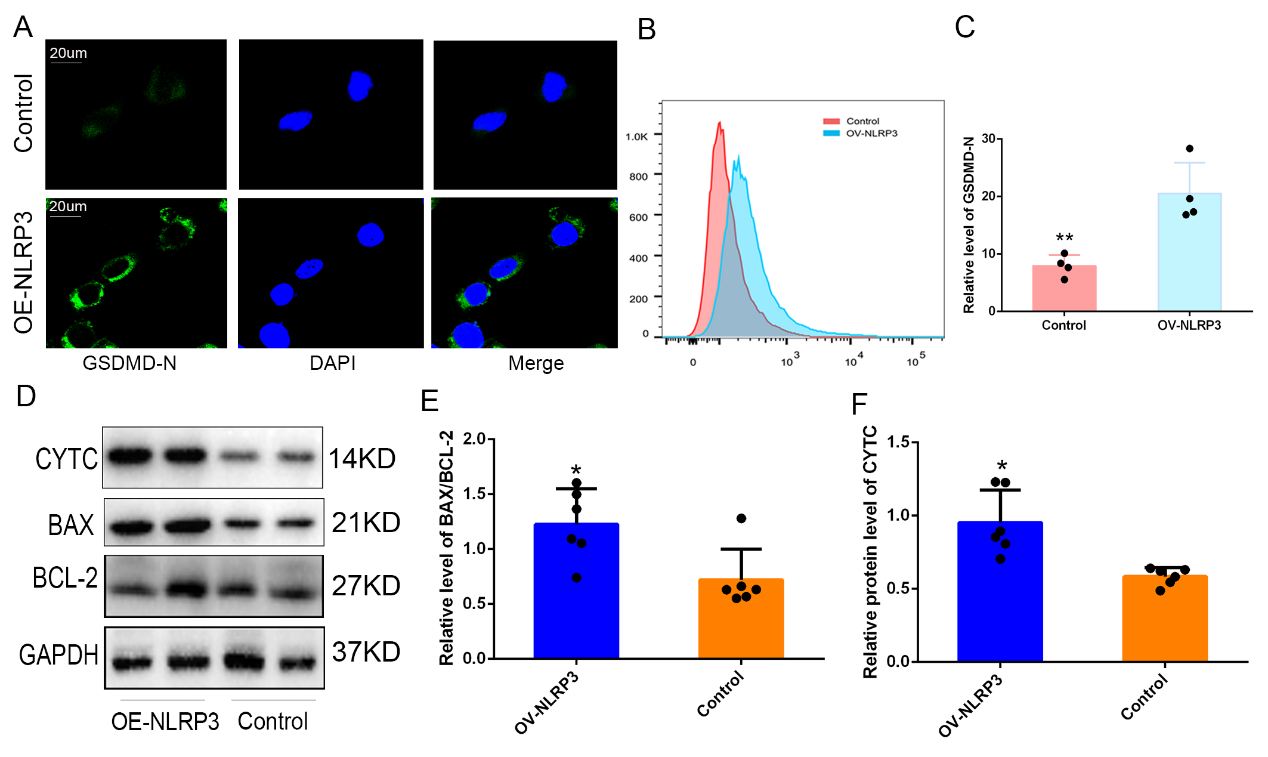


Figure s3: (A-C) Representative immunofluorescence images of GSDMD-N in control and OE-NLRP3 cells. Scale bar, 10μm, n=4. Quantitative analysis of flow cytometry showed that the level of GSDMD-N was significantly increased in OE-NLRP3 group. (D-F) Relative level of proteins in apoptotic pathway after overexpression NLRP3. These results suggested that overexpression of NLRP3 led to increased apoptosis in muscle cells, n=6. Data are shown as the mean ± SD, * means p < 0.05 vs. the control group. **means p < 0.01 vs. the control group.

Figure S4


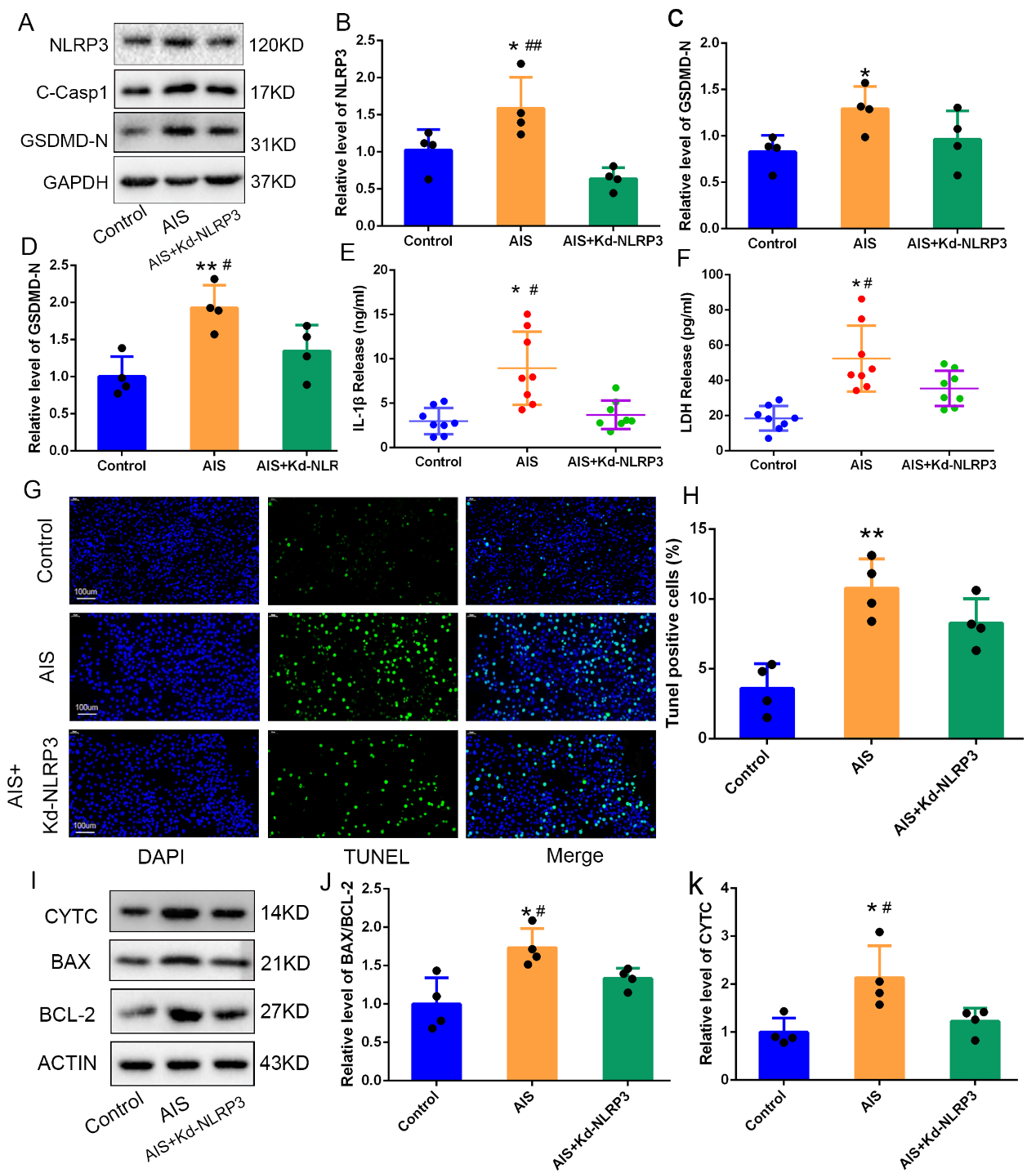


Figure s4: Knock down of NLRP3 ameliorated the pyroptosis in AIS primary muscle cells, while there was no obvious effect on apoptosis. (A-D) Protein levels of NLRP3, C-Caspase 1 and GSDMD-N in normal, AIS and AIS + Kd-NLRP3 groups. (E, F) Levels of LDH and IL-1β in the supernatant of primary muscle cell culture medium. (G, H) Representative images of TUNEL immunofluorescence staining in normal, AIS and AIS + Kd-NLRP3 primary muscle cells. Scale bar, 100μm. (I-K) Levels of pyroptosis pathway related proteins: CYTC and BAX/BCL-2 in normal, AIS and AIS + Kd-NLRP3 groups. Data are shown as the mean ± SD, * means p < 0.05 and ** means p < 0.01 vs. the control group. # means p<0.05 vs. the AIS + Kd-NLRP3 group.

Figure S5


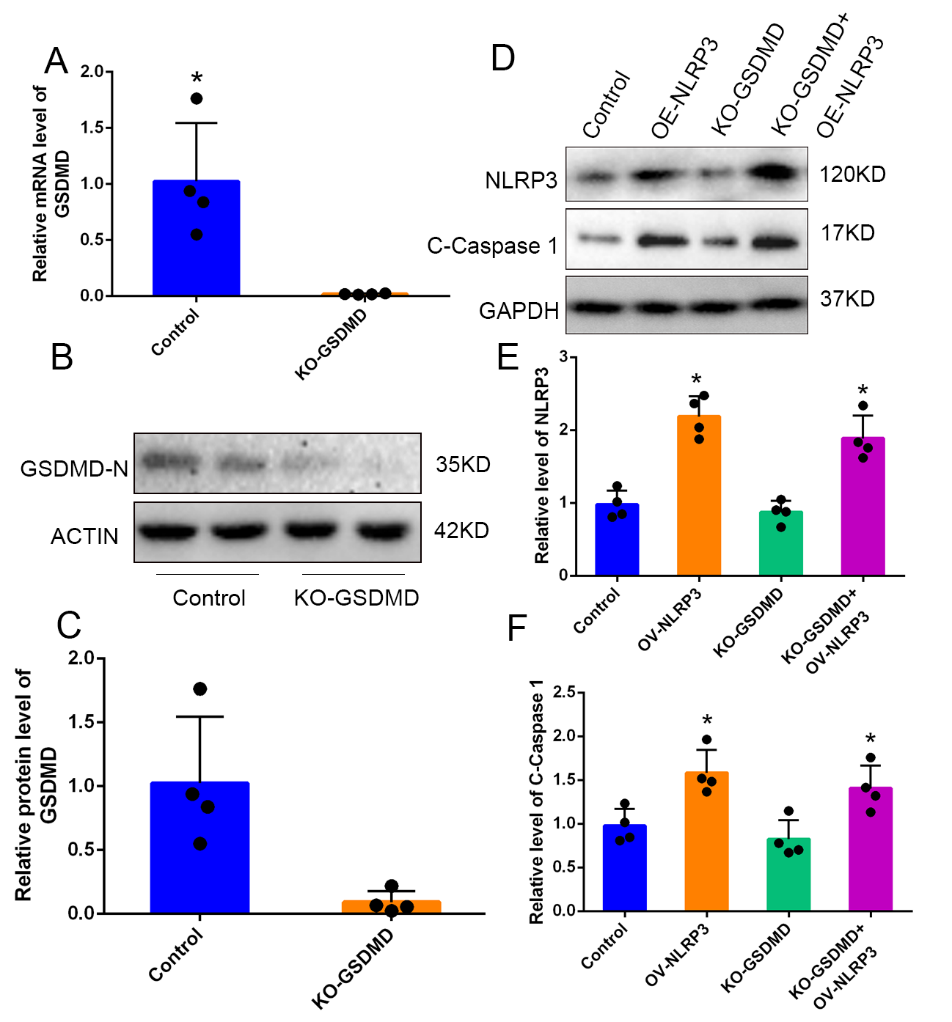


Figure s5: Expression of proteins in pyroptosis pathway after *GSDMD* knockout. (A-C) The mRNA and protein level of GSDMD after knockout of *GSDMD* gene by crispr-cas9 technique, n=4. (D-F) Protein level of NLRP3 and C-Caspase 1in c2c12 cells, n=4. Data are shown as the mean ± SD, * means p < 0.05 vs. the control group.

Figure S6


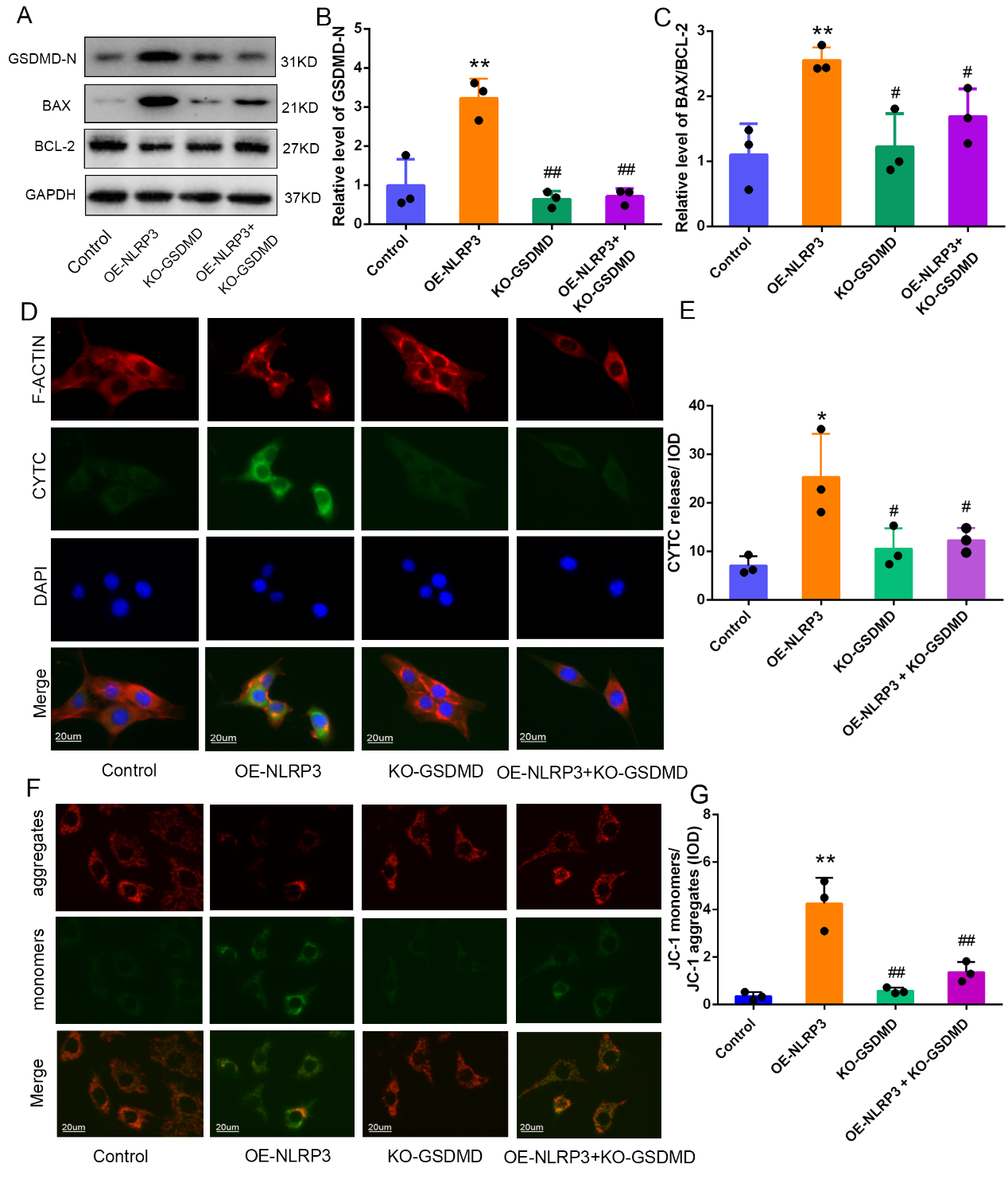


Figure s6: Knock out of the GSDMD gene inhibited apoptotic pathway which was activated by NLRP3. (A-C) Protein levels of GSDMD-N and BAX/BCL-2 in CT, OE-NLRP3, KO-GSDMD and KO-GSDMD + OE-NLRP3 groups separately. (D, E) Representative Immunofluorescence images showing CYTC release in CT, OE-NLRP3, KO-GSDMD and KO-GSDMD + OE-NLRP3 groups. Scale bar, 20μm. (F, G) Representative Immunofluorescence images of JC-1 fluorescent staining showing mitochondrial membrane potential in CT, OE-NLRP3, KO-GSDMD and KO-GSDMD + OE-NLRP3 groups. Scale bar, 20μm. Data are shown as the mean ± SD, * means p < 0.05 and ** means p < 0.01 vs. the control group, # means p<0.05 and ## means p < 0.01 vs. the OE-NLRP3 group.

Figure S7


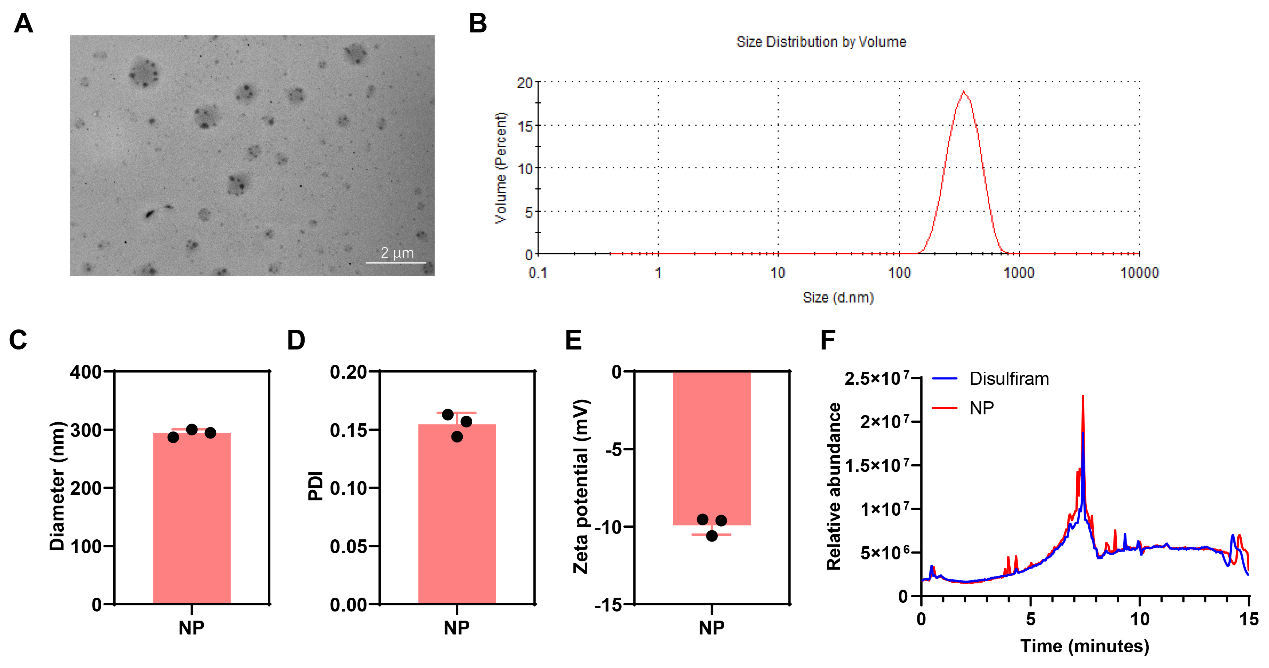


Figure s7: Physical and chemical properties of nanoparticles. (A) TEM shows the circular structure of NP. (B) Peak plot of particle size distribution of NP was detected by DLS. (C, D) Particle size and PDI values of NP, which were measured by DLS. (E) Zeta potential of NP. (F) LC-MS results showed the relative abundance of disulfiram and NP.
