# Supplementary material for: Critical role of NLRP3 in causing paravertebral muscle injury in adolescent idiopathic scoliosis
Source: Clin Transl Med. 2024 Feb 8;14(2):e1528. doi: 10.1002/ctm2.1528 (PMC10851084; doi:10.1002/ctm2.1528)
Supplement: Supplementary file 3 — Supplementary Tables [file CTM2-14-e1528-s001.docx]

**Table S1: Clinical data of enrolled patients in this study.**

| Items | AIS | control | P value |
| --- | --- | --- | --- |
| Number | 30 | 15 |  |
| Age | 12.6±3.5 | 15.2±4.2 | 0.15 |
| Gender | 14(boy) vs 16(girl) | 9(boy) vs 7(girl) |  |
| Main curve cobb angle | 58.5±12.4 |  |  |

Student's t-test was use to tested, when p value > 0.05, there was no significant difference in the two groups.

**Table S2:** **Primers for quantitative real-time PCR (qRT-PCR).**

| Gene |  | Primer sequences (5' to 3') |
| --- | --- | --- |
| NLRP3 | R  F | GCTTCTGGTTGCTGCTGAGGAC  AGGGATGAGAGTGTTGTGTGAAACG |
| IL-1β | R  F | TGCATTTGGATGTACAGATCG  CCCCCTCCCAAAAGAAGTAT |
| SMAD3 | R  F | GGCTCGCAGTAGGTAACTGG  CCCCAGAGCAATATTCCAGA |
| FOXO4 | R  F | TCGAGTTCTTCCATCCTGCT  TGTAACAGGTCCTCGGAAGG |
| GLUL | R  F | TGGCACAAAGCGACTGGATGAAC  GTGGAGGCCGACTTCTTGTATGC |
| GSDMD | R  F | CGATGGGAACATTCAGGGCAGAG  ACACATTCATGGAGGCACTGGAAC |
| RN18s | R  F | AGAAACGGCTACCACATCCA  CCCTCCAATGGATCCTCGTT |

**Table S3:** **Sequences of NLRP3 siRNA**

| Gene |  | sequences (5' to 3') |
| --- | --- | --- |
| siNLRP3 1 | sense | GCUUCAGGUGUUGGAAUUA |
| siNLRP3 2 | sense | UUAGACAACUGCAACCUCA |
| siControl | sense | GGCTCGCAGTAGGTAACTGG |

**Table S4: crRNA sequences of KO-GSDMD**

| crRNA | Target sequences (5' to 3') |
| --- | --- |
| crRNA 1 | TGCCCCCAGGACCATGCCAT |
| crRNA 2 | TGTCGTCGATGGGAACATTC |
| crRNA 3 | TCGTGGGGATGACCTGTTTG |
| crRNA 4 | CCACCAAAGCCGGAAGAAGA |
| crRNA 5 | GTTTCTCATCTGAGACGAGA |
